# Sculpting non-Hermitian light in the continuum

**DOI:** 10.1038/s41377-026-02450-y

**Published:** 2026-08-26

**Authors:** Xin Zhang, Zi-Lan Deng, Chaofan Zhang, Xiangping Li

**Affiliations:** 1 Guangdong Provincial Key Laboratory of Optical Fiber Sensing and Communications, Institute of Photonics Technology, College of Physics & Optoelectronic Engineering, Jinan University, Guangzhou, China; 2 College of Advanced Interdisciplinary Studies, National University of Defense Technology, Changsha, 410073 China

**Keywords:** Optical materials and structures, Optical physics

## Abstract

PT- or anti-PT-symmetric non-Hermitian photonic systems can exhibit responses such as phase transitions, slow light, and asymmetric modal evolution near an exceptional point (EP), but realizing a phase-transition process that satisfies anti-PT symmetry and passes through an EP on an integrated platform remains challenging. A smart research from Xiankai Sun’s group at the Chinese University of Hong Kong (CUHK) recently demonstrated that two quasi-BIC modes generate dissipative coupling to the same radiative continuum, enabling anti-PT phase transitions and EPs on a silicon-based integrated photonic platform without additional lossy materials or auxiliary lossy waveguides. This provides a new physical framework for exploring the intersection of BIC photonics and non-Hermitian photonics.

**Figure:**
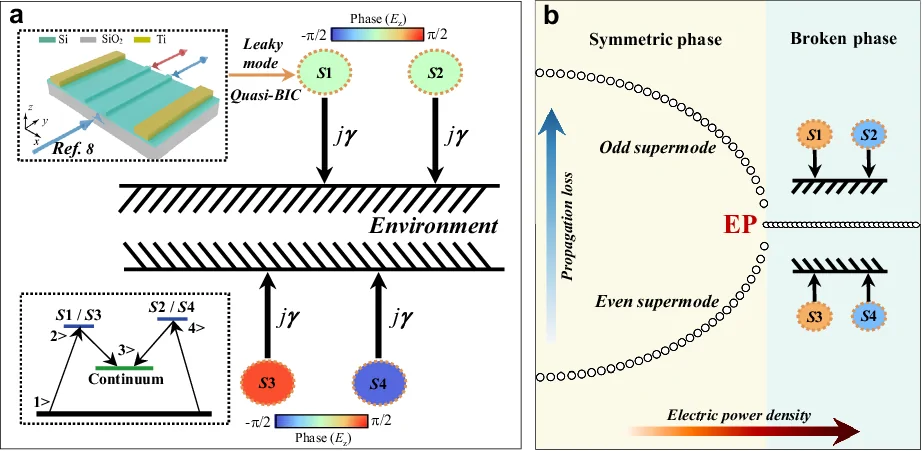

Non-Hermitian photonics expands the degrees of freedom for modal control in optical systems by introducing loss, gain, and environmental coupling^1–3^. Among them, PT-symmetric and anti-PT-symmetric systems are important platforms for studying non-Hermitian phase transitions and exceptional points (EPs). They can undergo phase transitions between symmetric and broken phases, with EPs appearing at the phase-transition boundaries where eigenvalues and eigenstates simultaneously coalesce. Owing to this unique non-Hermitian degeneracy, related systems have been used to explore functions such as slow light, enhanced sensing, asymmetric modal conversion, and on-chip non-Hermitian dynamical control.

For anti-PT photonic systems, the key issue is not only to introduce loss or dissipative coupling, but also to simultaneously satisfy the anti-PT-symmetry and EP conditions in experiments. Such systems usually need to maintain specific complex coupling relations while using controllable asymmetry to drive the system from the symmetric phase across an EP into the broken phase. However, in practical integrated platforms, the effective refractive index, propagation phase, structural parameters, and loss distribution are usually coupled with each other, making it difficult to independently control these conditions. Previously, anti-PT symmetry and its EP dynamics have been experimentally realized in flying atoms, optical fibers, passive nanophotonic structures, and integrated silicon photonic platforms^4–7^, but many schemes still rely on additional gain/loss distributions, auxiliary dissipative channels, or specific coupling structures. To address this issue, Sun and co-workers proposed and experimentally realized a binary quasi- Bound states in the continuum (BICs) integrated photonic system, using the radiative loss of quasi-BICs and the dissipative coupling generated through a shared continuum to realize anti-PT phase transitions and EP control on a silicon-based platform^8^.

BICs are special wave states whose eigenfrequencies lie within the radiative continuum but that can avoid outward radiation and remain spatially localized^9^. In photonics, BICs and their perturbed counterparts, quasi-BICs, have been widely exploited to realize high-Q resonances with Fano line shapes, promising form applications ranging from lasing, sensing, to nonlinear enhancement^9–11^. An ideal BIC corresponds to the complete suppression of radiative channels; when the structure deviates from the ideal condition, the resulting quasi-BIC reacquires tunable radiative loss, thereby coupling to the external continuum and allowing the mode to be externally excited, detected in the far field, and even indirectly dissipative coupled through a shared continuum^12^.

The finite radiative loss of quasi-BICs provides a new physical entry point for constructing anti-PT-symmetric systems. A single weakly confined rib waveguide can support a BIC at a specific width; once it deviates from this condition, the mode transforms into a quasi-BIC with finite radiative loss. When separated by an appropriate distance, the two quasi-BIC waveguides couple to the same radiative continuum and establish an indirect dissipative coupling through this shared radiative channel. As shown in Fig. 1a, the two quasi-BIC waveguides correspond to four bare states, *S*_1_–*S*_4_, with different phases or field distributions. These bare states are connected to the external environment through the shared radiative channel and combine to form odd and even supermodes. At a specific coupling phase, the composite system can simultaneously support a lossless supermode BIC and a lossy state. The corresponding anti-PT phase-transition process is shown in Fig. 1b. By further tuning the difference in the real part of the effective refractive index between the two quasi-BICs through the electric power density, the propagation losses of the odd and even supermodes can gradually approach each other, driving the system from the anti-PT-symmetric phase through an EP into the broken phase^8^.

**Fig. 1:**
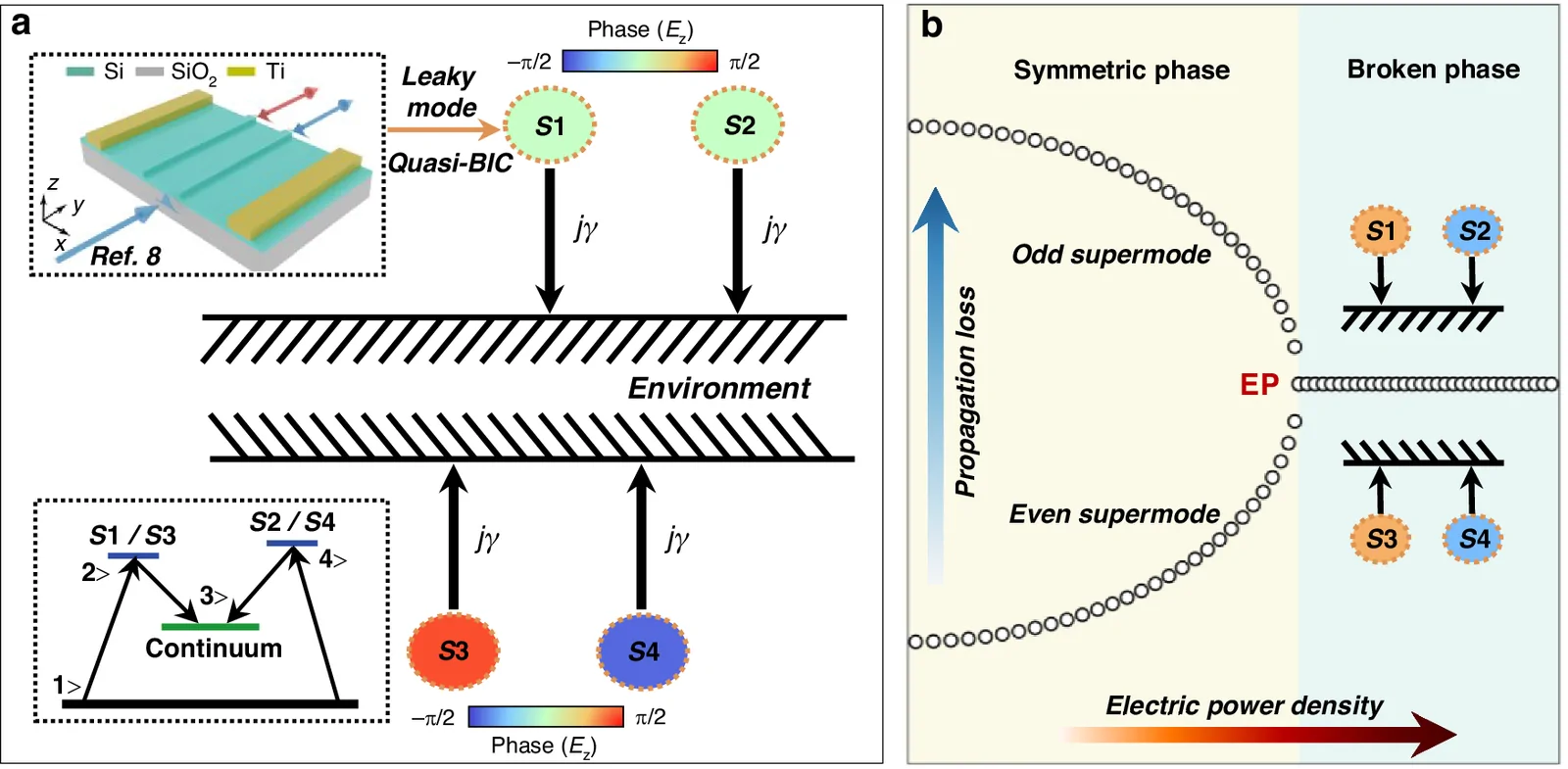
Dissipative coupling and anti-PT phase-transition mechanism in a binary quasi-BIC system. **a** The upper-left inset shows the weakly confined rib waveguide structure used to realize the quasi-BIC states. The lower-left inset shows the indirect coupling generated between two quasi-BIC states through a shared radiative continuum. *S*_1_–*S*_4_ denote four bare states corresponding to the two quasi-BIC waveguides under different phases or field distributions. The colors represent the phase distribution of (*E*_z_), and the black arrows indicate radiative coupling between the bare states and the external continuum. The four bare states form dissipative coupling through the shared continuum and further combine into odd and even supermodes. **b** Evolution of propagation loss under electrical-power-density tuning. The thermo-optic effect changes the difference in the real parts of the effective refractive indices between the two waveguides, causing the propagation losses of the odd and even supermodes to gradually approach each other and coalesce at the EP under specific conditions, thereby driving the system from the symmetric phase into the broken phase

Experimentally, a binary quasi-BIC structure was fabricated on an SOI platform and differential thermo-optic tuning was realized by using integrated titanium heaters. The results show that this system can realize an anti-PT phase transition and an EP on chip; even in the presence of fabrication deviations, the operating point can be reselected through wavelength and electrical power, yielding a slow-light response with a group index exceeding 40. Exceptional-line encircling experiments further demonstrate its potential for on-chip control of non-Hermitian dynamics^8^.

Related studies have previously realized BIC photonic circuits, observed dissipative coupled BICs generated by a shared continuum, and demonstrated dynamic EP encircling in anti-PT integrated photonic systems^7,11,13^; related theory has also proposed the formation mechanism of BICs in anti-PT systems^14,15^. The binary quasi-BIC system further shows that the finite radiative loss of quasi-BICs can in turn serve as a dissipative-coupling resource for constructing anti-PT symmetry.

Although this scheme demonstrates the role of quasi-BIC radiative loss in constructing anti-PT symmetry, there remains room for further development. The coupling phase in the shared radiative continuum depends on the operating wavelength, giving the EP condition a certain degree of wavelength selectivity; although thermo-optic tuning provides a stable experimental approach, it remains limited in terms of response speed and power consumption. In the future, combining this scheme with faster and lower-power on-chip tuning mechanisms, and extending the continuum-coupling scheme to multi-quasi-BIC modal systems, may further broaden the routes for realizing anti-PT phase transitions and EP dynamics in integrated non-Hermitian photonic devices. In this way, the finite radiative loss of quasi-BICs is not only a source of dissipation in open systems, but can also serve as a designable degree of freedom for constructing dissipative coupling.
